# Late-onset pulmonary complications among survivors of coronavirus Disease 2019

**DOI:** 10.12669/pjms.39.4.6302

**Published:** 2023

**Authors:** Mirza Muhammad Ayub Baig, Muhammad Adnan, Muhammad Usman Baig, Zohaib Ramzan

**Affiliations:** 1 Mirza Muhammad Ayub Baig, Department of Pulmonology, Sir Ganga Ram Hospital, Lahore, Pakistan; 2 Muhammad Adnan, Health Research Institute, National Institute of Health, HRI NIH Research Center FJMU, Lahore, Pakistan; 3 Muhammad Usman Baig, Ameer-ud-Din Medical College, Lahore, Pakistan; 4 Zohaib Ramzan Department of Pulmonology, Sir Ganga Ram Hospital, Lahore, Pakistan

**Keywords:** COVID-19, Dyspnea, Pulmonary fibrosis, SARS-CoV-2, Tachycardia, Post-COVID

## Abstract

**Objectives:**

To assess the late-onset pulmonary complications among survivors of coronavirus disease 2019.

**Methods:**

The cross-sectional analytical study was conducted in the department of Pulmonology, Sir Ganga Ram Hospital Lahore between October 2020 and March 2021. Total 288 patients visiting the hospital 12-week after recovery from COVID-19 enrolled using convenience sampling. After excluding patients (n=61) with a history of previous respiratory symptoms before the development of COVID-19, data from 227 patients was subjected to final analysis. Chest X-ray (CXR) was used to evaluate lung condition.

**Results:**

Participation of middle-aged adults (54.6%) was higher than older (38.3%) and young adults (7.0%). The percentage of males was 55.5% and smokers was 29.1%. Dyspnea was the most common complication as 80.0% patients had moderate to severe dyspnea while chronic cough was 78.0% and lung fibrosis (LF) was 13.2%. The chances of LF increased with the rise in age (p-value 0.033). However, the distribution of LF was similar between males and females. The frequency of lung fibrosis in smokers was 3-time higher than among non-smokers (24.2 vs. 8.7%; p-value 0.003). The patients with LF were more dependent on O2 as compared to the patients without LF (p-value < 0.001). The frequency of tachycardia was significantly different between patients with and without LF (all p-values < 0.05).

**Conclusion:**

LF is a common late-onset pulmonary complication of COVID-19 and is associated with old age, smoking, O_2_ dependency, tachycardia, and severe dyspnea.

## INTRODUCTION

Severe acute respiratory syndrome Coronavirus-2 (SARS-CoV-2) affected a huge population of the world.^1^ The SARS-CoV-2 affects different systems of the body including the nervous, gastrointestinal, cardiovascular, and respiratory systems.^2^ The respiratory system involvement may manifest as upper respiratory tract infection (URTI) or pneumonia. The COVID-19 patients without pneumonia develop URTI and present in the clinic with mild symptoms of URTI.^3^

Pneumonia is the most deleterious sequelae of coronavirus disease 2019 (COVID-19) that leads to respiratory failure.^4^ However despite high mortality, a considerable number of COVID-19 patients with pneumonia are being managed and discharged from hospitals.

Whereas, some patients do not feel well three to four weeks after discharge from the hospital and present with different complaints at various levels of health facilities. A considerable number of patients fully recovered from COVID-19 are still facing dyspnea on exertion.

Various studies evaluated the number of patients whose exercise capacity was reduced after recovery from SARS-CoV.^5^ A study suggested that cardiac injury continued despite recovery from COVID-19.^6^ Similarly, many patients treated in the intensive care unit (ICU) on invasive or noninvasive ventilation still require oxygen at home. A study elaborated long-lasting effects on lung function with the severity of pneumonia in patients of Middle East Respiratory Syndrome (MERS) due to MERS-CoV infection 2015 outbreak in Korea, and showed persistent radiological changes with impaired lung function and reduced vital capacity after one year.^7^ Therefore, the study aimed to assess the late-onset pulmonary complications among survivors of COVID-19. As the follow-up of these patients and addressing the complications is an important part of the management of COVID-19.

## METHODS

The study was approved by the COVID-19 Research Committee of Fatima Jinnah Medical University Lahore Pakistan vide letter No.5679-89/F.J. dated 18th September 2020. Written informed consent was taken from all patients. The cross-sectional analytical study was carried out at the department of Pulmonology, Sir Ganga Ram Hospital Lahore Pakistan between October 2020 and March 2021. Total 288 patients of age 14-90 years, and visiting hospital outpatient department 12-week after recovery from COVID-19 were enrolled using convenience sampling. After excluding patients (n=61), data from 227 patients was subjected to final analysis. Persistent parenchymal changes on High resolution CT-scan (HRCT) i.e., Ground-Glass opacities (GGO), reticular changes and traction bronchiectasis of interstitial pneumonia are consistent histopathologically with lung fibrosis (LF).^8^ HRCT is preferred medium to assess the parenchymal changes in lungs. However, study by Abo-Hedibah et al showed that the results of Chest X-Ray (CXR) and HRCT are very close in COVID pneumonia.^9^ So, we decided to use CXR due to easy access and low cost.

### Inclusion criteria:

Patients of age 14-90 years, visiting hospital outpatient department 12-week after recovery from COVID-19 were enrolled.

### Exclusion criteria:

All patients (n=61) with a history of previous respiratory symptoms before the development of COVID-19 were excluded.

### Data collection procedure:

All data was collected upon enrollment in the study. Age, gender, smoking status, O_2_ dependency, and Brixia score at the time of COVID-19 were noted from their files. Oxygen saturation (SpO_2_) at rest and after 6-minute walk test (6MWT), pulse rate (PR) at rest and after 6MWT, Medical Research Council (MRC) dyspnea scale, O_2_ dependency, CXR score, presence of LF and chronic cough 12-week after recovery from COVID-19 were estimated by the researcher.

### Operational definitions:

Reverse transcription-polymerase chain reaction (RT-PCR) positive result on nasopharyngeal swab sample defined as COVID-19.^10^ Two consecutive RT-PCR negative results or remained afebrile for at least three days defined as COVID-19 survivor.^11^ SpO_2_<97.0% at rest and <94.0% after 6MWT defined as oxygen desaturation.^12^ PR>100BPM at rest and after 6MWT defined as tachycardia.^13^ The MRC dyspnea scale was used to calculate dyspnea score.^14^ LF labeled radiologically (moderate to severe BRIXIA score) as sequelae of COVID-19 pneumonia leading to persistent parenchymal changes (GGO, reticular opacities and traction bronchiectasis) predominantly in the middle and lower radiological zone.^8^

### BRIXIA Score:

We used BRIXIA CXR score for quantifying persistent parenchymal changes in COVID-19. In the first step, each lung is divided into three equal radiological zones by two imaginary lines on each side. In the, second step a score is designated to each zone based on lung abnormalities as follow:

Score=0 if no lung abnormalities, score=1 if GGO, score=2 as GGO and reticular opacities, and score=3 as GGO, reticular opacities plus traction bronchiectasis. Score of all six zones was summed to assess the radiological severity of the disease. Radiologic score 0 categorized as normal, 1-6 as mild, 7-12 as moderate, and 13-18 as severe.^15^

### Statistical analysis:

Statistical Package for Social Sciences (SPSS) version 22 was used for analysis. Categorical variables were reported using frequency (percent). Continuous variables were categorized into groups and reported using frequency (percent). Chi-square test was used to compare the frequencies between groups. P-value ≤0.05 was considered significant. Logistic regression was used to measure correlation.

## RESULTS

Among COVID-19 survivors (n=227), the percentage of males (55.5%) was higher than females (44.5%). The characters of the sample population like age, O_2_ dependency, smoking and Brixia score at the time of COVID-19 are summarized in Table-I.

**Table-I:** Characteristics of the study population at the time of COVID-19 (n=227).

		Frequency	Percent
Age (years)	≤35(young)	16	7.0
	36-55(middle-aged)	124	54.6
	≥ 56(older)	87	38.3
Gender	Male	126	55.5
	Female	101	44.5
Smoking	No	161	70.9
	Yes	66	29.1
O_2_ dependency (L/min)	0	06	2.6
	1-2	10	4.4
	3-5	20	8.8
	6-10	102	44.9
	>10	89	39.2
Brixia CXR score	0(normal)	0	0.0
	1-6(mild)	03	1.3
	7-12(moderate)	123	54.2
	13-18(severe)	101	44.5

After 12-week of recovery from COVID-19, the parameters of the sample population like SpO_2_, pulse rate etc. are summarized in Table-II. There was a strong correlation (R-value = 0.797) between Brixia CXR score at time of COVID and oxygen requirement at the time of COVID-19. Similarly, there was a correlation (R-value=0.463) observed between CXR score 12 weeks after COVID and MRC dyspnea after 12 weeks. LF was seen in 13.2% of the patients and chronic cough with variable intensity was observed in 78.0% of the patients. Table-II.

**Table-II:** Pulmonary complications 12-week after recovery from COVID-19 (n=227).

		Frequency	Percent
SpO_2_ at rest (%)	<97.0	191	84.1
	≥97.0	36	15.9
SpO_2_ after 6MWT (%)	<94.0	145	63.9
	≥94.0	82	36.1
PR at rest(BPM)	≤100(normal)	210	92.5
	>100(tachycardia)	17	7.5
PR after 6MWT(BPM)	≤100(normal)	94	41.4
	>100(tachycardia)	133	58.6
MRC dyspnea scale	1(mild)	44	19.4
	2-3(moderate)	104	45.8
	4-5(severe)	79	34.8
O_2_ dependency(L/min)	0	136	59.9
	1-2	65	28.6
	3-5	16	7.0
	6-10	07	3.1
	>10	03	1.3
CXR score	0(normal)	42	18.5
	1-6(mild)	155	68.3
	7-12(moderate)	27	11.9
	13-18(severe)	03	1.3
Lung fibrosis	No	197	86.8
	Yes	30	13.2
Chronic cough	No	50	22.0
	Yes	177	78.0

The chances of LF increased with the increase in age (p-value=0.033). The smokers had a 3-time higher proportion of LF than non-smokers (24.2 vs 8.7%; p-value=0.003). The patients with LF were more dependent on O_2_ than the patients without LF (p-value <0.001). The frequencies of O_2_ desaturation at rest and after 6MWT, tachycardia at rest and after 6MWT, the severity of dyspnea and chronic cough were significantly different between patients with and without LF. We Divided patients into two groups based on diagnosis of LF 12-Week post recovery from COVID. We then compared different parameters of the two groups at the time of COVID and 12-week post covid in Table-III. Additionally, three patients developed pneumothorax, two patients developed empyema and another two patients developed deep vein thrombosis (DVT).

**Table-III:** Comparison between patients with and without LF (n=227).

Lung Fibrosis - Yes (n=30)			Lung Fibrosis - No (n=197)		
			Frequency	Percent	Frequency	Percent	
At the time of COVID-19	Age(years)	≤35	0	0.0%	16	100.0%	0.033
		36-55	14	11.3%	110	88.7%	
		≥56	16	18.4%	71	81.6%	
	Gender	Female	13	12.9%	88	87.1%	0.891
		Male	17	13.5%	109	86.5%	
	Smoking	No	14	8.7%	147	91.3%	0.003
		Yes	16	24.2%	50	75.8%	
	O_2_ dependency	0	0	0.0%	06	100.0%	<0.001
		1-2	0	0.0%	10	100.0%	
		3-5	1	5.0%	19	95.0%	
		6-10	4	3.9%	98	96.1%	
		>10	25	28.1%	64	71.9%	
12-week after recovery from COVID-19	SpO_2_at rest(%)	<97	30	15.7%	161	84.3%	0.006
		≥ 97	0	0.0%	36	100.0%	
	SpO_2_after 6MWT(%)	<94	28	19.3%	117	80.7%	0.001
		≥94	2	2.4%	80	97.6%	
	PR at rest(BPM)	≤100	24	11.4%	186	88.6%	0.014
		>100	6	35.3%	11	64.7%	
	PR after 6MWT(BPM)	≤100	4	4.3%	90	95.7%	0.002
		>100	26	19.5%	107	80.5%	
	MRC dyspnea scale	1	0	0.0%	44	100.0%	<0.001
		2-3	4	3.8%	100	96.2%	
		4-5	26	32.9%	53	67.1%	
	O_2_ dependency	0	0	0.0%	136	100.0%	<0.001
		1-2	15	23.1%	50	76.9%	
		3-5	11	68.8%	05	31.3%	
		6-10	3	42.9%	04	57.1%	
		>10	1	33.3%	02	66.7%	
	CXR Score	0	0	0.0%	42	100.0%	<0.001
		1-6	0	0.0%	155	100.0%	
		7-12	27	100.0%	0	0.0%	
		13-18	3	100.0%	0	0.0%	
	Chronic cough	No	13	26.0%	37	74.0%	0.005
		Yes	17	9.6%	160	90.4%	

## DISCUSSION

COVID-19 is known for its deleterious health effects leading to the respiratory failure. Its severe form represents viral pneumonia from SARS-CoV-2 infection leading to acute respiratory distress syndrome (ARDS), and its clinical manifestations can be observed as a blend of viral pneumonia and ARDS.^16^ We know that COVID-19 patients may present with primary symptoms of gastrointestinal and cardiovascular diseases. However, it manifests with respiratory tract infection in the majority of the patients.^2^ In the beginning, the healthcare professionals focused on early diagnosis of COVID-19 and treatment of respiratory failure. However, despite recovery from respiratory failure, some patients died because of pulmonary complications. Therefore, the present study focused on late-onset pulmonary complications among patients who recovered from moderate to severe COVID-19. The results showed that a significant number of patients developed LF, chronic cough and were dependent on O_2_ at home. The patients who had severe COVID-19 and were dependent on high flow O_2_ still faced respiratory complications even at three-month follow-up. These findings are in agreement with the results reported in other studies. Hall et al. reported that mean HR at follow-up was 111+17BPM, 40.0% of the patients had dyspnea, and 32.0% had LF manifesting persistent interstitial parenchymal changes. However, the study population also included 27.5% of patients who required mechanical ventilator support at the time of COVID-19.^17^ Similarly, mean PR at follow-up 105.3±13.4BPM and 30.0% LF was observed in the present study, where none of the included patients required ventilator support. Wu et al. also evaluated COVID-19 patients at three-month follow-up and found that mild dyspnea was present in 81.0% patients and moderate in 6.0% patients.^18^ Differently, the respective frequencies of mild, moderate, and severe dyspnea were 19.4%, 45.8%, and 34.8% in the present study.

We assessed the radiological severity of the disease using CXR rather than HRCT to reduce expenses as Masood et al describe X-Ray findings in COVID Pneumonia.^19^ Similarly Rashid et al elaborate the role of CXR in COVID-19 pneumonia, discouraging the wastage of resources.^20^ The patients having normal SpO_2_ at rest after recovery from COVID-19 develop dry cough of variable intensity, aggravated at night or early morning and similar in character to bronchial asthma.^21^ Similarly chronic cough, developed in 78.0% of patients, was the most common complication in the present study. Few patients also developed pneumothorax after recovery from COVID-19. Only two patients developed DVT in the present study. The low incidence of DVT might be due to sound knowledge of prophylactic anticoagulation among physicians in treating COVID-19.

LF and chronic cough were the most common pulmonary complications after recovery from acute disease. The misery of patients is not relieved by surviving the acute phase of COVID-19 and patients are still facing the consequences of COVID-19 in the form of respiratory failure and O_2_ dependency creating a significant economic burden.

### Limitations:

The present study includes an CXR rather than HRCT scan because of economical constraint. Spirometry could not be performed due to the risk of reinfection to the patients.

## CONCLUSIONS

LF is a common late-onset pulmonary complication of COVID-19 and is associated with old age, smoking, O_2_ dependency, tachycardia, and severe dyspnea. These findings suggest that COVID-19 is related to pulmonary complications even after recovery. It indicates much improved drugs needed for prompt treatment of SARS-CoV-2 infection associated pneumonia.

### Authors Contribution:

**MMAB:** designed the study, wrote original draft, critically reviewed the manuscript and approved the final version to be published.

**MA:** performed analysis and interpretation of data, critically reviewed and revised the manuscript and approved the final version to be published.

**MUB:** performed collection of data, critically reviewed the manuscript and approved the final version to be published.

**ZR:** performed collection of data, critically reviewed the manuscript.

All authors have approved the final version to be published and take responsibility for the content and similarity index of the manuscript.
